# Impact of the SARS-CoV-2 pandemic on the diagnosis of primary cutaneous melanoma at a University Hospital in Rio de Janeiro^☆^

**DOI:** 10.1016/j.abd.2022.04.001

**Published:** 2022-08-26

**Authors:** Carlos Baptista Barcaui, Carla Jorge Machado, Juan Piñeiro-Maceira

**Affiliations:** a Department of Dermatology, Faculty of Medical Sciences, Universidade do Estado do Rio de Janeiro, Rio de Janeiro, RJ, Brazil; b Department of Preventive and Social Medicine, Faculty of Medicine, Universidade Federal de Minas Gerais, Rio de Janeiro, RJ, Brazil; c Department of Pathology, Universidade Federal do Rio de Janeiro, Rio de Janeiro, RJ, Brazil

Dear Editor,

Melanoma is the most severe type of skin cancer and its incidence has been increasing worldwide, although its mortality rate has remained stable and it has shown a decreasing trend in some countries in recent decades.^1^ Early diagnosis and advances in the treatment of progressive disease are likely responsible for this stabilization in mortality rates.^2^ From a financial point of view, cutaneous melanoma represents a critical burden for Brazil, and the cost of the disease varies according to the Health system (public versus private) and disease stage at the time of the diagnosis. Expenditures on patients with advanced disease can be up to 34-fold (Brazilian Unified Health System) or 270-fold (private health sector) higher than what is required to treat early-stage disease.^3^

Due to the health restrictions imposed by the SARS-Cov2 pandemic, access to health services in the city of Rio de Janeiro was impaired, especially in 2020. As a result, there was practically a suspension in the follow-up of high-risk patients and screening tests for melanoma at Hospital Universitário Pedro Ernesto (HUPE). Measuring the impact of reduced skin cancer screening in this period on the worsening of the prognosis of patients with cutaneous melanoma is a challenge, but a necessary indicator for health services. The aim of the present study was to evaluate the impact of the SARS-CoV-2 pandemic on the diagnosis of primary cutaneous melanoma in the Dermatology Service of a university hospital in the city of Rio de Janeiro.

## Methods

A cross-sectional, descriptive and analytical observational study was carried out on cases of melanoma diagnosed at the Dermatology Service of the HUPE. Cases diagnosed as primary cutaneous melanoma that was confirmed on histopathological examination, in the period between 2012 and 2021, were included. The impact of the SARS-CoV-2 pandemic on the diagnosis of primary cutaneous melanoma was evaluated by comparing the number of diagnosed cases and the annual average of tumor thickness measured in millimeters during this period.

The association between two qualitative variables was verified through the test of difference in proportions using Fisher's adjustment for small numbers. Poisson regression was used to estimate the incidence ratios and 95% confidence intervals. The statistical analysis was performed using Stata/SE software, version 12.0 for Mac.

## Results

In the last ten years, since the outpatient treatment of pigmented lesions was started at the Dermatology Service of HUPE, 91 new cases of cutaneous melanoma were diagnosed in 89 patients, with one patient having two asynchronous melanomas and another patient with two synchronous melanomas. Of the 91 assessed tumors, 24% (22) were *in situ* lesions and 75% (69) were invasive tumors with a mean thickness of 1.04 mm (0.10‒5.72 mm), with a predominance of 47% (42) of lesions with Clark II invasion level. Table 1 shows the distribution of the Breslow thickness medians observed between the years 2012 and 2021. The mean number of cases diagnosed between the years 2013 and 2019 was 9.8 cases/year. In 2020, a decrease of 49.0% (5) was observed in the number of diagnosed cases. In the year 2021, there were 11 diagnosed cases. In the first trimester of 2021 alone, the same number of melanoma cases were diagnosed as along the entire year 2020. The findings in Table 2 indicate that melanoma incidence rates decreased when comparing the 2018/2019 and 2020/2021 biennia, ranging from 3.15 cases per 1,000 appointments to 1.34 cases per 1,000 in 2020/2021. The incidence ratios indicated that the 2020 and 2021 rates were, respectively, 72% and 80% lower when compared to the 2018/2019 rate. The lowest rate was accompanied by the greater severity of the cases, which can be corroborated by Clark's invasion levels with a clear tendency to advance in 2020/2021 compared to 2018/2019. Thus, it can be observed that in half of the cases in 2020/2021, the levels of invasion were IV or V, whereas this percentage was 5% in 2018/2019.

**Table 1:** Number of cases of cutaneous melanoma diagnosed in the Dermatology service of HUPE between March 2012 and December 31, 2021, mean age, and median Breslow thickness of invasive cases.

		Patient age^a^	Breslow thickness (mm)^a^
Year	Number of Cases	Median (p25; p75)	Median (p25; p75)
2012 (Mar‒Dec)	6	64 (58; 75)	0.44 (0.00; 0.57)
2013	11	60 (58; 71)	0.41 (0.00; 0.93)
2014	6	62 (57; 77)	0.00 (0.00; 0.16)
2015	9	63 (47; 65)	0.50 (0.31; 0.70)
2016	13	60 (56; 67)	0.23 (0.00; 0.77)
2017	9	75 (58; 85)	0.45 (0.30; 1.12)
2018	11	73 (52; 80)	0.30 (0.00; 0.50)
2019	9	65 (53; 73)	0.60 (0.20; 0.60)
2020	6	83 (71; 85)	1.40 (0.00; 3.30)
2021	11	72 (57; 81)	0.50 (0.30; 2.60)
Total	91	65 (57; 76)	0.40 (0.05; 0.83)

Notes p25: 25th percentile; p75: 75th percentile.

^a^Median and percentiles used because the Shapiro-Wilks test indicated a lack of normality for age (p = 0.049) and for Breslow thickness (p < 0.001).

**Table 2:** Summary table of the differences found between the years 2018/2019; 2020; 2021 (January to March) related to cases of melanoma diagnosed in the Dermatology service of HUPE.

Analyzed variables and statistics	2018/2019	2020/2021
Number of melanoma cases	20	16
Number of appointments	6.349	11,912
Incidence rates (per 1,000 appointments)	3,15	1.34
Incidence ratios (95%CI)^a^	1,00	0.44 (0.27;0.73)
	‒	p = 0.001
Breslow thickness in mm: Median (p25; p75)	0,40 (0,15; 0,60)	0.80 (0.30; 2.90)
		p = 0.502^b^
Clark's invasion level, n (%)		
I	4 (20,0)	3 (18.7)
II or III	15 (75,0)	5 (31.3)
IV or V	1 (5,0)	8 (50.0)
		p = 0.041^c^
Age in years: Mean (SD)	65,0 (18,0)	72.0 (13.5)
		p = 0.205^d^

Notes: SD, Standard Deviation; p25, 25th percentile; p75, 75th percentile.

a Estimates of incidence ratio, 95% confidence intervals, and p-value obtained by Poisson regression.

b p-value based on nonparametric Mann-Whitney test for comparison of independent medians (the Shapiro-Wilks test indicated absence of normality: p < 0.001).

c p-value based on nonparametric Cuzick trend test.

d P-value based on Student's *t* test for comparison of independent means (the Shapiro-Wilks test did not indicate an absence of normality: p = 0.088).

## Discussion

As recently demonstrated through a growth rate model, the probability of increased thickness and progression to a more advanced disease stage is 21%, 29%, and 45% with a one, two, and a three-month delay in melanoma diagnosis, respectively.^4^

Considering the restrictions imposed by the health crisis, one can infer that the SARS-CoV-2 pandemic is the most probable justification for a lower monthly rate of diagnosed cases in the period after the beginning of the pandemic and higher Breslow thicknesses than in the last ten years.

The worldwide incidence of melanoma has been increasing in recent decades, and mortality rates seem to show a tendency towards stabilization. One of the main reasons for this stabilization is early diagnosis. Although recent scientific research carried out in the United States of America has proposed the interruption of campaigns for the early detection of skin cancer as a way to reduce the number of diagnosed melanomas, this does not seem to be a prudent strategy to be adopted.^5^ When one compares the number of melanomas diagnosed at HUPE in the previous decade (2002‒2011; n = 71) an increase of around 10% can be observed. However, as the present data demonstrate, there was a reduction of more than 50% in the number of diagnosed cases in 2020 and the cases that were detected were at a more advanced stage. Similarly to what happened with other types of cancer and what was observed with the diagnosis of melanoma in Italy, during the beginning of the SARS-CoV-2 pandemic, a significant reduction was observed in the number of diagnosed cases of cutaneous melanoma, which is potentially associated with increased morbidity, mortality, and financial costs.^6^, ^7^, ^8^, ^9^, ^10^, ^11^, ^12^, ^13^

The impact of the delay in the diagnosis caused by the SARS-CoV-2 pandemic on the survival of patients with melanoma and the costs involved in treating the disease in more advanced stages should be the subject of future studies.

## Conclusion

In the first year of the SARS-CoV-2 pandemic (2020) a significant reduction was observed in the number of diagnosed cases of primary cutaneous melanoma and the average thickness of the tumors was greater when compared to the cases diagnosed in the previous eight years.

## Financial support

None declared.

## Authors' contributions

Carlos Baptista Barcaui: Approval of the final version of the manuscript; design and planning of the study; drafting and editing of the manuscript; collection, analysis, and interpretation of data; effective participation in research orientation.

Carla Jorge Machado: Statistical analysis; critical review of the manuscript.

Juan Piñeiro-Maceira: Intellectual participation in the propaedeutic and/or therapeutic conduct of the studied cases; critical review of the literature; critical review of the manuscript.

## Conflicts of interest

None declared.
